# Diagnostic Performance of Neural Network-Based Artificial Intelligence in the Detection and Classification of Pediatric Astrocytoma: A Systematic Review

**DOI:** 10.7759/cureus.83543

**Published:** 2025-05-05

**Authors:** Nermin Ezat Elbakry Abdelmahdy, Essra Tajelsir Abd Alnour Suliman, Fatima Mahmoud Osman Mohmed, Nazik Siddig Ali Elamin, Belgees Altigani Hamza Yousif, Nazik Abbas Mohammed Ahmmed

**Affiliations:** 1 Paediatrics, Ashford and St. Peter’s Hospital, Chersty, GBR; 2 Paediatrics, Royal Surrey County Hospital, Guildford, GBR; 3 General Medicine, Sharourah General Hospital, Sharourah, SAU; 4 General Medicine, East and North Hertfordshire NHS Trust, Stevenage, GBR; 5 Paediatrics, Najran Armed Forces Hospital, Ministry of Defense, Health Services, Najran, SAU; 6 Paediatrics, Ateika Medical Health Center-First Health Cluster In Riyadh, Riyadh, SAU

**Keywords:** artificial intelligence, diagnostic accuracy, neural networks, pediatric astrocytoma, posterior fossa tumors

## Abstract

Pediatric posterior fossa tumors, particularly astrocytomas, pose significant diagnostic challenges due to their histological diversity and overlapping imaging features. Conventional methods relying on radiologists' interpretations are prone to subjectivity and variability. Neural network-based artificial intelligence (AI) has emerged as a promising tool to enhance diagnostic accuracy. This systematic review evaluates the diagnostic performance of AI models in pediatric astrocytoma detection and classification, synthesizing evidence from six studies to assess strengths, limitations, and clinical applicability. Following Preferred Reporting Items for Systematic Reviews and Meta-Analyses (PRISMA) guidelines, a systematic search of PubMed, Excerpta Medica database (Embase), Web of Science, and Scopus identified 291 records. After deduplication, 102 studies underwent title/abstract screening, 26 advanced to full-text review, and six met inclusion criteria. Studies were included if they evaluated neural network-based AI for pediatric posterior fossa tumor diagnosis and reported quantitative performance metrics. Risk of bias was assessed using Quality Assessment of Diagnostic Accuracy Studies-2 (QUADAS-2), and findings were synthesized narratively due to methodological heterogeneity. Included studies demonstrated high diagnostic accuracy for AI models, with area under the receiver operating characteristic curve (AUROC) values exceeding 0.99 and classification accuracies up to 95%. AI frequently outperformed radiologists, particularly in distinguishing histologically similar tumors. Innovations like 3D texture analysis and multi-parametric Magnetic resonance imaging (MRI) integration enhanced performance. However, the small number of included studies, limited sample sizes, and retrospective designs limit the generalizability of these findings. Methodological concerns, such as high risk of bias in patient selection and use of subjective reference standards, were also noted. Neural network-based AI shows transformative potential in pediatric astrocytoma diagnostics, offering superior accuracy and efficiency compared to conventional methods. Nonetheless, clinical translation requires addressing these methodological limitations, along with enhancing dataset diversity, ensuring prospective validation, and considering ethical implications. Future research should prioritize multi-center trials, explainable AI frameworks, and integration of multi-modal data to bridge the gap between experimental models and real-world clinical practice.

## Introduction and background

Pediatric posterior fossa tumors, including astrocytomas, represent a critical subset of childhood cancers with significant morbidity and mortality [1]. These tumors, such as pilocytic astrocytomas, medulloblastomas, and ependymomas, account for approximately 60% to 70% of all pediatric brain malignancies, often presenting with nonspecific symptoms that complicate timely diagnosis [2]. Accurate and early differentiation of tumor types is paramount, as treatment strategies and prognoses vary substantially. For instance, pilocytic astrocytomas, while typically low-grade, require precise surgical planning to avoid neurological deficits, whereas medulloblastomas demand aggressive multimodal therapy due to their malignant potential [3]. Conventional diagnostic workflows rely heavily on magnetic resonance imaging (MRI), supplemented by histopathological examination. However, MRI interpretation remains subjective, with inter-observer variability among radiologists and challenges in distinguishing histologically similar tumors based on imaging features alone [4]. This diagnostic uncertainty can delay treatment initiation, particularly in resource-constrained settings where access to subspecialized neuroradiologists is limited. 

In recent years, artificial intelligence (AI), particularly neural network-based algorithms, has emerged as a potentially valuable adjunct in medical imaging, offering the capacity to enhance diagnostic accuracy, reduce human error, and standardize interpretations [5]. Deep learning models, such as convolutional neural networks (CNNs), excel at identifying complex patterns in imaging data, including subtle texture variations and spatial relationships that may elude human observers [6]. These capabilities are increasingly being explored in pediatric neuro-oncology, where the integration of multi-parametric MRI data, such as diffusion-weighted imaging, perfusion metrics, and spectroscopy, may support more precise tumor characterization [7]. Despite these advances, the application of AI in pediatric populations remains underexplored compared to adult cohorts, partly due to the rarity of pediatric tumors, ethical constraints in data collection, and the unique biological behavior of childhood neoplasms. Existing studies on AI for pediatric brain tumors are fragmented, with heterogeneity in methodologies, sample sizes, and outcome measures, complicating efforts to synthesize actionable insights for clinical translation [8, 9]. 

This systematic review addresses these gaps by comprehensively evaluating the diagnostic performance of neural network-based AI systems in pediatric astrocytomas, with a focus on posterior fossa tumors. By synthesizing evidence from existing studies, this review aims to assess the strengths and limitations of current AI models, compare their performance against conventional diagnostic methods, and identify barriers to clinical implementation.

## Review

Methodology

The methodology for this systematic review was designed to comprehensively evaluate the diagnostic performance of neural network-based AI in pediatric astrocytomas, adhering to the Preferred Reporting Items for Systematic Reviews and Meta-Analyses (PRISMA) guidelines [10]. The process encompassed five key stages: search strategy development, study selection, data extraction, risk of bias assessment, and data synthesis. Each stage was executed with rigorous protocols to ensure reproducibility, minimize bias, and address the review’s objectives.

Search strategy and information sources

A systematic literature search was conducted across four electronic databases: PubMed, Excerpta Medica database (Embase), Web of Science, and Scopus. The search strategy combined Medical Subject Headings (MeSH) terms and free-text keywords related to three core concepts: (1) pediatric posterior fossa tumors (e.g., “pediatric astrocytoma,” “medulloblastoma,” “ependymoma”), (2) artificial intelligence (e.g., “neural network,” “deep learning,” “machine learning”), and (3) diagnostic performance (e.g., “diagnostic accuracy,” “sensitivity,” “area under the curve”). Boolean operators (AND/OR) were used to refine the search, and filters were applied to restrict results to human studies published in English. Review articles, editorials, and gray literature, including conference proceedings and preprint repositories, were excluded to prioritize peer-reviewed evidence.

Study selection criteria

Studies were included if they investigated neural network-based AI models for diagnosing or classifying pediatric posterior fossa tumors, including astrocytomas, and reported quantitative performance metrics such as accuracy, sensitivity, specificity, or area under the receiver operating characteristic curve (AUROC). Exclusion criteria encompassed non-AI methodologies (e.g., statistical models without machine learning), adult or animal studies, review articles, and studies lacking sufficient methodological detail. Two independent reviewers (NEEA & FMOM) from the list of authors screened titles and abstracts, followed by full-text assessments of potentially eligible articles. Discrepancies were resolved through consensus discussions or by consulting a third author (BAHY) as a tiebreaker, all of whom were co-authors of this review.

Data extraction process

A standardized data extraction template of Microsoft Excel (Microsoft Corporation, Redmond, WA, USA) was developed to systematically capture information from each included study. Key variables encompassed study characteristics (author, year, location, and design), population details, AI algorithms, comparators, and outcomes. Two reviewers independently extracted data, cross-verifying entries for consistency.

Risk of bias assessment

The methodological quality of included studies was evaluated using the Quality Assessment of Diagnostic Accuracy Studies-2 (QUADAS-2) tool [11], which assesses risk of bias and applicability concerns across four domains: patient selection, index test, reference standard, and flow/timing. Each domain was rated as “low,” “high,” or “unclear” risk based on predefined criteria. For example, studies with small sample sizes were flagged for high risk of patient selection bias, which could lead to inflated diagnostic performance metrics, such as overestimated AUROC values. Studies using expert consensus as a reference standard were rated with low risk, though subjectivity in these standards was noted. A narrative discussion was provided to explain how these biases might affect AI model performance, particularly in terms of robustness and generalizability. Disagreements between reviewers were resolved through iterative discussions, ensuring consensus on final ratings.

Data synthesis and analysis

Given the heterogeneity in study designs, AI algorithms, and outcome measures, a narrative synthesis was employed rather than a meta-analysis. Findings were organized thematically to address the review’s objectives: (1) comparing AI performance against radiologists or traditional methods, (2) evaluating advancements in imaging techniques (e.g., 3D texture features, multi-parametric MRI), and (3) identifying barriers to clinical implementation. Quantitative results, such as AUROC values and classification accuracies, were tabulated for direct comparison. Subgroup analyses were conducted to explore trends, such as the superior performance of models integrating multi-modal data versus those relying on single modalities. Limitations, including retrospective designs and variability in reference standards, were critically discussed to contextualize the evidence.

Ethical considerations

As this review synthesized data from previously published studies, ethical approval was not required. However, the review adhered to principles of transparency and intellectual integrity by citing all sources accurately and avoiding selective reporting. Potential conflicts of interest were mitigated by excluding studies funded exclusively by AI software developers. The PRISMA checklist was completed to ensure methodological rigor and accountability throughout the review process.

Results

Study Selection Process

**Figure 1:**
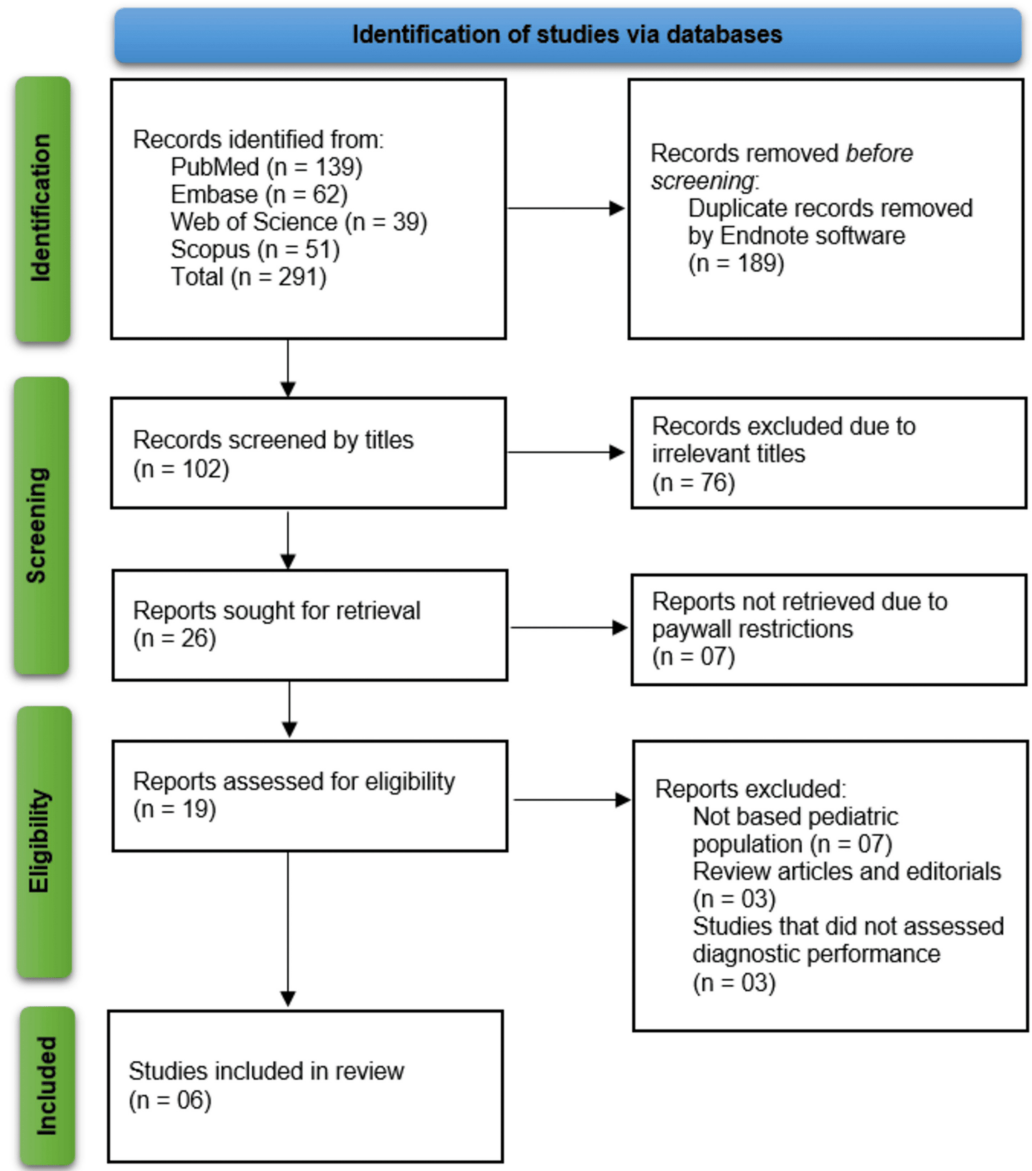
An overview of study identification, screening, eligibility assessment, and inclusion, following PRISMA guidelines PRISMA: Preferred Reporting Items for Systematic Reviews and Meta-Analyses; Embase: Excerpta Medica database

The study selection process began with a comprehensive search across four major databases: PubMed (n = 139), Embase (n = 62), Web of Science (n = 39), and Scopus (n = 51), resulting in a total of 291 records. After removing 189 duplicate records using EndNote software (Clarivate, Philadelphia, PA, USA), 102 unique records remained for title screening. Out of these, 76 were excluded due to irrelevant titles, leaving 26 reports for retrieval. However, seven of these could not be accessed due to paywall restrictions. Nineteen full-text reports were assessed for eligibility, of which 13 were excluded (seven for not focusing on the pediatric population, three for being review articles or editorials, and three for not evaluating diagnostic performance). Ultimately, six studies [12-17] met the inclusion criteria and were retained for qualitative synthesis in the systematic review (Figure 1).

Summary of the Included Studies 

The systematic review included six studies [12-17] published between 1997 and 2020, all investigating the diagnostic performance of neural network-based AI in pediatric posterior fossa tumors, with a focus on astrocytomas. The studies were conducted in the United States [12, 16, 17] and the United Kingdom [13-15]. Study designs were predominantly retrospective diagnostic studies, with one diagnostic accuracy study [14] and one model development and validation study [16]. 

Sample sizes varied significantly across studies. The largest study by Quon et al. [12] included 816 pediatric patients (617 with tumors and 199 controls), while smaller studies such as Fetit et al. [15], Bidiwala and Pittman [16], and Arle et al. [17] had samples of 48, 33, and 33 patients, respectively. Two studies [13, 14] did not specify sample sizes, which may limit the generalizability of their findings. The populations across all studies consisted of pediatric patients with posterior fossa tumors, including pilocytic astrocytomas, medulloblastomas, and ependymomas. 

Various AI algorithms were employed, with neural networks being the most common. Quon et al. [12] utilized a modified ResNeXt-50-32x4d multitask classifier, achieving an AUROC > 0.99 for tumor detection and 92% classification accuracy. Orphanidou-Vlachou et al. [13] compared a probabilistic neural network (PNN) with linear discriminant analysis (LDA), reporting up to 93.3% accuracy using T2-weighted MRI texture features. Fetit et al. [15] demonstrated that 3D texture features improved diagnostic accuracy by 19% over 2D features when used with neural networks. Earlier studies by Bidiwala and Pittman [16] and Arle et al. [17] also employed neural networks, with the latter achieving 95% accuracy when combining magnetic resonance spectroscopy (MRS), MRI, and clinical data. 

Comparators varied, ranging from radiologist interpretations [12, 16, 17] to traditional machine learning methods like LDA [13] and texture-based analyses [15]. Key outcomes included diagnostic accuracy, AUROC, and classification performance metrics such as F1 scores. Notably, AI models frequently outperformed human radiologists in classification tasks, as seen in Quon et al. [12] and Arle et al. [17]. 

The studies highlighted the potential of AI in improving diagnostic precision for pediatric brain tumors, though limitations such as small sample sizes and retrospective designs were noted. Advances in deep learning and multi-parametric MRI integration, as demonstrated by Grist et al. [14] and Quon et al. [12], suggest promising directions for future research (Table 1).

**Table 1:** Summary and key findings of studies included in this systematic review AI: artificial intelligence; AUROC: area under the receiver operating characteristic curve; F1 SCORE: harmonic mean of precision and recall; RESNEXT-50-32X4D: a variant of ResNeXt deep learning model (residual network with aggregated transformations); PNN: probabilistic neural network; LDA: linear discriminant analysis; PC: principal components; PCA: principal component analysis; MRI: magnetic resonance imaging; LOOCV: leave-one-out cross-validation; AUC: area under the curve; CT: computed tomography; MRS: magnetic resonance spectroscopy; PNET: primitive neuroectodermal tumor; PFT: posterior fossa tumor

Author	Publishing year	Location	Study design	Population type	Sample Size	AI algorithms	Comparator	Outcomes	Key findings
Quon et al., [12]	2020	United State	Retrospective diagnostic study	Pediatric patients with posterior fossa tumors and controls	816 (617 with tumors, 199 controls)	Modified ResNeXt-50-32x4d multitask classifier	4 radiologists	AUROC for tumor detection, classification accuracy, F1 score	Tumor detection AUROC > 0.99; classification accuracy 92%, F1 score 0.80; outperformed 2 of 4 radiologists in classification accuracy and F1 score
Orphanidou‐Vlachou et al., [13]	2014	United Kingdom	Retrospective study	Children with posterior fossa tumours (pilocytic astrocytomas, medulloblastomas, ependymomas)	Not stated	PNN, LDA, PCA	LDA, clinical interpretation	Classification accuracy of PNN and LDA using texture features from T1- and T2-weighted MRI scans	PNN classifier achieved up to 93.3% (T2) and 90% (T1) accuracy on LOOCV. Combined T1+T2 PCs reached 85.8%. LDA accuracy was lower. Texture features provided novel diagnostic insights.
Grist et al., [14]	2020	United Kingdom	Diagnostic accuracy study	Pediatric brain tumour patients	Not specified	Machine learning classifiers	Expert qualitative review (standard)	Diagnostic performance using diffusion and perfusion MRI features	Combined features from diffusion and perfusion imaging achieved >80% predictive precision in classification
Fetit et al., [15]	2015	United Kingdom	Retrospective observational study (inferred)	Pediatric patients with brain tumors (medulloblastoma, pilocytic astrocytoma, ependymoma)	48	Neural network and five other supervised classifiers	2D texture features vs 3D texture features	AUC, overall classification accuracy	Neural network trained with 3D features improved AUC by 12% and classification accuracy by 19% over 2D features; statistically significant improvements for four classifiers based on McNemar’s test
Bidiwala and Pittman, [16]	2004	United State	Model development and validation study	Children with posterior fossa tumors (PFTs)	33	Neural network using clinical and imaging (CT and MRI) data	Neuroradiologists	Accuracy of tumor classification	Correct classification in 85.7% (complete data) and 72.7% (incomplete data); outperformed neuroradiologists in both cases
Arle et al., [17]	1997	United State	Retrospective diagnostic study	Pediatric patients with posterior fossa tumors	33	Neural network (trained on MRS, MRI features, and clinical data)	The neuroradiologist's prediction based on MRI data only	Diagnostic accuracy in predicting tumor type (astrocytoma, PNET, or ependymoma/other)	The neural network using all data achieved 95% accuracy; the neuroradiologist achieved 73%. Networks using only spectroscopy had the lowest accuracy (58%). Accuracy improved with additional clinical and imaging data (up to 95%).

Risk of Bias Assessment Results

The risk of bias assessment revealed varying levels of methodological rigor across the six included studies. Quon et al. [12] and Fetit et al. [15] demonstrated low risk of bias across all domains, reflecting strong methodological quality and appropriate comparator usage. Orphanidou‐Vlachou et al. [13] showed low risk in the index test domain but had unclear risks in patient selection, reference standard, and flow due to insufficient reporting. Grist et al. [14] also presented low risk in the index test and reference standard domains, but unclear risk in patient selection and flow. In contrast, Bidiwala and Pittman [16] and Arle et al. [17] were judged to have a high risk of bias in patient selection and applicability due to small sample sizes and limited methodological detail, though their index tests remained low risk. These findings highlight the variability in study design quality among the available literature on AI-based diagnosis of pediatric astrocytoma (Table 2).

**Table 2:** Risk of bias assessment using QUADAS-2 tool QUADAS-2: Quality Assessment of Diagnostic Accuracy Studies-2

Study	Patient selection	Index test	Reference standard	Flow and timing	Applicability concerns
Quon et al., [12]	Low	Low	Low	Low	Low
Orphanidou‐Vlachou et al., [13]	Unclear	Low	Unclear	Unclear	Low
Grist et al., [14]	Unclear	Low	Low	Unclear	Low
Fetit et al., [15]	Low	Low	Low	Low	Low
Bidiwala and Pittman [16]	High	Low	Unclear	Unclear	High
Arle et al., [17]	High	Low	Low	Unclear	High

Discussion

This systematic review synthesized evidence from six studies evaluating the diagnostic performance of neural network-based AI in pediatric posterior fossa tumors, with a focus on astrocytomas. The findings collectively demonstrate that AI models, particularly deep learning architectures and neural networks, achieve high diagnostic accuracy, often surpassing human radiologists in classification tasks. For instance, Quon et al. [12] reported an AUROC > 0.99 for tumor detection and 92% classification accuracy using a modified ResNeXt-50 model, outperforming two of four radiologists. Similarly, Arle et al. [17] achieved 95% accuracy by integrating MRI, spectroscopy, and clinical data, compared to 73% accuracy by neuroradiologists. These results underscore the potential of AI to augment diagnostic precision in pediatric neuro-oncology.

Notably, advancements in imaging analysis techniques, such as 3D texture features [15] and multi-parametric MRI [14], further enhanced diagnostic performance. For example, 3D texture features improved classification accuracy by 19% over 2D features, highlighting the importance of leveraging spatial information. However, variability in sample sizes and study designs introduced heterogeneity in outcomes. While Quon et al. [12] and Grist et al. [14] utilized large or multi-site datasets, smaller studies [13, 16, 17] faced limitations in generalizability due to sample sizes as low as 33 patients. Retrospective designs dominated the included studies, raising concerns about selection bias and reproducibility in prospective clinical settings.

The findings align with broader trends in AI research for medical imaging, where neural networks consistently demonstrate superior performance in tasks such as tumor detection and classification. For example, a meta-analysis by Liu et al. [18] on AI in neuro-oncology reported pooled sensitivities and specificities exceeding 90% for glioma classification, corroborating the high AUROC values observed in Quon et al. [12]. However, pediatric-specific studies remain scarce, and most literature focuses on adult populations. Pediatric tumors, such as pilocytic astrocytomas, exhibit distinct imaging and molecular profiles compared to adult gliomas, necessitating tailored AI models. The emphasis on posterior fossa tumors in this review fills a critical gap, as these tumors account for 60-70% of pediatric brain malignancies [19, 20].

In contrast, comparisons with unrelated imaging fields, such as mammography and pulmonary nodule detection [21, 22], may be less directly applicable to pediatric neuro-oncology due to the unique characteristics of pediatric brain tumors. Comparatively, studies in adult cohorts often integrate genomic data (e.g., IDH mutation status) with imaging, a practice less common in pediatric research due to ethical and logistical challenges. For instance, Zhou et al. [20] achieved 89% accuracy in adult glioma subtyping by combining MRI features with transcriptomic data, whereas pediatric studies like Arle et al. [17] relied on clinical and conventional imaging data. This disparity underscores the need for multi-modal pediatric datasets to enhance model robustness. Furthermore, the outperformance of AI over radiologists in classification tasks mirrors findings in mammography [21] and pulmonary nodule detection [22], suggesting a broader trend of AI augmenting human expertise.

However, discrepancies exist in the magnitude of AI’s superiority. While Quon et al. [12] reported significant improvements over radiologists, Orphanidou-Vlachou et al. [13] observed more modest gains (93.3% vs. 85.8% accuracy for PNN vs. LDA). These differences may stem from variations in input data quality, annotation protocols, or radiologist expertise. For example, the use of standardized imaging protocols in Quon et al. [12] likely reduced variability, whereas older studies [17] relied on legacy imaging equipment, potentially introducing noise. To further enhance consistency and reliability in future research, there is a clear need for standardization in imaging protocols and AI model reporting across studies. Additionally, while future directions are well articulated, clearer prioritization of these directions would help guide the next steps in AI development for pediatric neuro-oncology.

Strengths and Limitations of the Included Studies

The included studies exhibit several methodological strengths. Quon et al. [12] and Grist et al. [14] utilized multi-institutional datasets, enhancing external validity and reducing site-specific biases. The integration of advanced imaging modalities, such as diffusion-weighted MRI [14] and 3D texture analysis [15], demonstrated the value of harnessing multi-parametric data. Additionally, the use of objective metrics like AUROC and F1 scores provided transparent performance evaluations, aligning with best practices in AI research [23].

However, significant limitations temper these strengths. Small sample sizes in older studies (e.g., n=33 in Arle et al. [17]) increase the risk of overfitting, limiting model generalizability. While modern studies like Quon et al. [12] mitigated this with larger cohorts, two studies [13, 14] did not report sample sizes, undermining reproducibility. Retrospective designs, prevalent across all studies, introduce selection bias and limit causal inferences. For instance, training models on historical data may not account for evolving imaging technologies or tumor subtypes. Ethical constraints in pediatric data collection, such as concerns over consent and privacy, may further limit large-scale validation efforts and contribute to the small sample sizes seen in these studies.

Another critical limitation is the lack of external validation. None of the included studies tested their models on independent, geographically diverse cohorts, a cornerstone of robust AI validation [24]. Furthermore, the reliance on subjective reference standards (e.g., radiologist interpretations) in Bidiwala & Pittman [16] and Arle et al. [17] introduces measurement bias. In contrast, studies employing expert consensus [12] or quantitative biomarkers [15] produced more reliable ground truths. Finally, the reliance on narrative synthesis instead of meta-analysis, while understandable due to data variability, limits the strength of the conclusions drawn and should be considered a constraint in evaluating the overall effectiveness of AI models in pediatric neuro-oncology.

Implications for Clinical Practice

The integration of AI into clinical practice holds transformative potential for pediatric neuro-oncology. AI could serve as a decision-support tool, particularly in resource-limited settings with scarce subspecialty expertise. For example, models like Quon et al.’s [12] ResNeXt-50 could expedite tumor detection, reducing delays in diagnosis and treatment initiation. Additionally, AI’s ability to discern subtle texture features [13] may aid in differentiating histologically similar tumors, such as pilocytic astrocytomas and ependymomas, which require distinct therapeutic approaches.

However, several barriers impede immediate clinical adoption. First, the "black-box" nature of neural networks complicates interpretability, a concern highlighted in recent FDA guidelines for AI-based devices [25]. Clinicians may hesitate to trust AI predictions without explainable visualizations, such as gradient-weighted class activation maps (Grad-CAMs). Second, ethical considerations, including data privacy and algorithmic bias, must be addressed. Pediatric datasets are inherently small and may underrepresent rare tumor subtypes or diverse populations, risking biased predictions. Third, regulatory hurdles necessitate rigorous validation through prospective trials. The current evidence, derived from retrospective studies, meets TRIPOD Level 4 criteria (prediction model development), but Level 1 evidence (impact studies assessing clinical outcomes) is lacking [26]. To facilitate clinical implementation, AI models will need regulatory approvals from agencies such as the FDA or European Medicines Agency (EMA), requiring extensive clinical trial data. Clinician training will also be essential to ensure trust in AI predictions and their integration into clinical workflows. Moreover, a cost analysis will be necessary to assess the financial feasibility of implementing AI tools in pediatric oncology settings.

Future Research Directions

To translate AI from research to clinical utility, a phased roadmap of short- and long-term goals is essential. In the short term, pilot studies and prospective trials across diverse institutions are essential to validate AI models in real-world settings. These trials should adhere to standards like the DECIDE-AI framework [27], ensuring transparency and reproducibility. Simultaneously, emphasis should be placed on developing interpretable models, using techniques like attention mechanisms or saliency maps, to promote clinician trust and adoption. Combining imaging with genomic, clinical, and outcome data could enhance predictive accuracy. For example, incorporating methylation profiles of pediatric tumors [28] may improve subtype classification. In the long term, the integration of AI into clinical workflows will require not only technical validation but also structural investment in infrastructure, clinician training, and ethical oversight. Collaborative efforts between AI developers, clinicians, and policymakers are needed to establish guidelines for data sharing, bias mitigation, and equitable access. Initiatives like the Federated Learning approach [29] could enable model training on decentralized datasets while preserving patient privacy. Assessing the economic impact of AI implementation will be vital for healthcare systems. For instance, reducing diagnostic delays could lower long-term treatment costs, but upfront investments in AI infrastructure must be justified.

## Conclusions

This systematic review underscores the promising yet preliminary role of neural network-based AI in diagnosing pediatric astrocytomas. Current evidence demonstrates high diagnostic accuracy, often surpassing traditional methods; however, small sample sizes, retrospective designs, and lack of external validation warrant cautious interpretation. The integration of advanced imaging techniques, multi-modal data, and explainable AI frameworks provides a foundation for future progress. Collaborative efforts across disciplines, including clinicians, data scientists, and policymakers, are essential to advance this field responsibly. As research evolves, carefully designed prospective studies and robust ethical frameworks will be critical to determining the true clinical utility of AI in pediatric neuro-oncology.
